# Non-vitamin K antagonist oral anticoagulants versus warfarin for the prevention of spontaneous echo-contrast and thrombus in patients with atrial fibrillation or flutter undergoing cardioversion: A trans-esophageal echocardiography study

**DOI:** 10.1371/journal.pone.0191648

**Published:** 2018-01-23

**Authors:** Yun Gi Kim, Jong-Il Choi, Mi-Na Kim, Dong-Hyuk Cho, Suk-Kyu Oh, Hyungdon Kook, Hee-Soon Park, Kwang No Lee, Yong-Soo Baek, Seung-Young Roh, Jaemin Shim, Seong-Mi Park, Wan Joo Shim, Young-Hoon Kim

**Affiliations:** Division of Cardiology, Department of Internal Medicine, Korea University College of Medicine and Korea University Medical Center, Seoul, Republic of Korea; Providence VA Medical Center, UNITED STATES

## Abstract

Spontaneous echo-contrast (SEC) and thrombus observed in trans-esophageal echocardiography (TEE) is known as a strong surrogate marker for future risk of ischemic stroke in patients with atrial fibrillation (AF) or atrial flutter (AFL). The efficacy of non-vitamin K antagonist oral anticoagulants (NOAC) compared to warfarin to prevent SEC or thrombus in patients with AF or AFL is currently unknown. AF or AFL patients who underwent direct current cardioversion (DCCV) and pre-DCCV TEE evaluation from January 2014 to October 2016 in a single center were analyzed. The prevalence of SEC and thrombus were compared between patients who received NOAC and those who took warfarin. NOAC included direct thrombin inhibitor and factor Xa inhibitors. Among 1,050 patients who were considered for DCCV, 424 patients anticoagulated with warfarin or NOAC underwent TEE prior to DCCV. Eighty patients who were anticoagulated for less than 21 days were excluded. Finally, 344 patients were included for the analysis (180 warfarin users vs. 164 NOAC users). No significant difference in the prevalence of SEC (44.4% vs. 43.9%; *p* = 0.919), dense SEC (13.9% vs. 15.2%; *p* = 0.722), or thrombus (2.2% vs. 4.3%; *p* = 0.281) was observed between the warfarin group and the NOAC group. In multivariate analysis, there was no association between NOAC and risk of SEC (odds ratio [OR]: 1.4, 95% CI: 0.796–2.297, *p* = 0.265) or thrombus (OR: 3.4, 95% CI: 0.726–16.039, *p* = 0.120). In conclusion, effectiveness of NOAC is comparable to warfarin in preventing SEC and thrombus in patients with AF or AFL undergoing DCCV. However, numerical increase in the prevalence of thrombus in NOAC group warrants further evaluation.

## Introduction

Atrial fibrillation (AF) is a prevalent disease that affects 1–2% of the general population. It is associated with increased risk of ischemic stroke and impaired quality of life [1–3]. Direct current cardioversion (DCCV), either electrical or pharmacological, is considered as an initial therapy to convert AF to sinus rhythm, especially in symptomatic patients [4]. However, DCCV is associated with increased risk of ischemic stroke during peri-DCCV period. Such risk might exceed 5% if adequate anticoagulation is not given [5–7]. Current guidelines recommend a minimum of 3 weeks of anticoagulation before DCCV followed by a minimum of 4 weeks of anticoagulation after DCCV [8, 9]. Absence of thrombus in left atrium (LA) and left atrial appendage (LAA) in trans-esophageal echocardiography (TEE) evaluation might significantly reduce the duration of adequate anticoagulation before DCCV [10].

Before the arrival of non-vitamin K antagonist oral anticoagulants (NOAC), therapeutic anticoagulation with warfarin before and after DCCV has been the mainstay of standard care. NOAC has proven to have equivalent or better efficacy compared to warfarin for the prevention of ischemic stroke in patients with AF [11–14]. In addition, NOAC is as effective as warfarin in preventing ischemic stroke in patients undergoing DCCV for AF [4, 15]. Nevertheless, in ENSURE-AF trial, stroke occurred in 5 patients in the edoxaban group (n = 1,095) and in 11 patients in the enoxaparin-warfarin group (n = 1,104), with an overall event rate of 0.73% [15]. In X-VeRT trial, event rates for stroke during peri-DCCV period were 0.2% in the rivaroxaban group and 0.4% in the warfarin group, with an overall event rate of 0.27% [4]. These extremely low event rates virtually limit statistical power to discriminate the antithrombotic efficacies of NOAC and warfarin in patients with AF undergoing DCCV.

Spontaneous echo-contrast (SEC) observed in TEE is considered as a pre-stage phenomenon of fibrin-rich red thrombus, a predominant form of thrombus observed in LA or LAA of patients with AF [16]. According to SPAF-III study, SEC was observed in 55% of patients with non-valvular AF [17]. SEC is a strong predictor of thrombus formation and future ischemic stroke events in AF patients [16, 18]. In addition to blood stasis, alteration of blood characteristics favoring coagulation is also related to SEC [19, 20]. Since SEC is considered as pre-stage phenomenon of red thrombus in AF and is more prevalent than thrombus or ischemic stroke event, SEC can be used as an outcome endpoint to facilitate the discrimination of antithrombotic efficacies of warfarin and NOAC. Therefore, the objective of this study was to compare the efficacies of warfarin and NOAC for the prevention of SEC or thrombus in patients with AF undergoing DCCV.

## Methods

### Patients

Patients with AF or atrial flutter (AFL) who were considered for DCCV from January 2014 to October 2016 in Korea University Anam Hospital were retrospectively analyzed. Inclusion criteria were as follows: (i) diagnosis of AF or AFL in patients aged over 19 years, (ii) available TEE data within 1 month before DCCV, and (iii) patients who were anticoagulated with NOAC or warfarin for at least 3 weeks before TEE evaluation. There was no specific exclusion criteria. Patients who were prescribed with aspirin or clopidogrel were excluded. The protocol of this study was consistent with the ethical guidelines of the 2008 Helsinki Declaration. Institutional Review Board of Korea University Anam Hospital ensured appropriate ethical and bioethical conduct and approved this specific study. Written informed consent was waived due to its retrospective nature. Patient records and information were anonymized prior to analysis.

### Direct current cardioversion

All DCCV procedures were performed by biphasic direct current shock. Patients were sedated with either midazolam or propofol under intensive monitoring. Energy delivered for DCCV ranged from 50 to 200 Joules. Continuous electrocardiogram (usually lead II) was recorded and monitored by trained medical doctors throughout the procedure. Procedural success was determined by continuous electrocardiogram monitoring or 12-lead electrocardiogram.

### Anticoagulation

Currently available NOACs (dabigatran, rivaroxaban, apixaban, and edoxaban) were all included in this study. Patients who were prescribed with reduced dose of NOAC were also included in the analysis. Reduced doses for each individual NOAC are as follows: (i) dabigatran: 110 mg twice daily, (ii) rivaroxaban: 15 mg or 10 mg once daily, (iii) apixaban: 2.5 mg twice daily, and (iv) edoxaban: 30 mg once daily. Selection of specific NOAC and its dose was based on treating physician’s discretion. For patients who were anticoagulated with warfarin, international normalized ratio (INR) was measured on the same day or one day prior to DCCV. Warfarin dosing and frequency of INR measurement were based on treating physician’s experience and discretion. Time in therapeutic range was calculated as follows: (number of INR measurements in 1.8–3.2/total INR measurements)*100. Time of INR > 1.8 was calculated by the following formula: (number of INR measurements above 1.8/total INR measurements)*100. NOAC and warfarin were maintained for at least 4 weeks after the DCCV. In subgroup analysis, patients prescribed with reduced dose of NOAC and full dose NOAC were analyzed separately.

### TEE

All TEE evaluations were performed by physicians dedicated in the field of cardiac imaging. Patients were sedated with midazolam before inserting the TEE probe. After obtaining routine views (high esophageal 0°, 45°, 60°, and 120° views for LA, left ventricle [LV], right chambers, and valves), LAA was evaluated using at least three different views (high esophageal 0°, 45°, 60°, or 120° views). Thorough evaluation of LA and LAA was performed to discover any evidence of SEC or thrombus. During LAA imaging, pulsed Doppler velocities of forward (emptying) and backward (filling) LAA flow were also recorded. All TEE evaluations were accompanied by trans-thoracic echocardiography (TTE) evaluations. LA size and LV ejection fraction were measured.

### Outcome endpoints

The prevalence rate of SEC, degree of SEC, and thrombus were compared between the warfarin group and the NOAC group. Diagnosis of SEC and thrombus was made before the conceptualization of this study based on the decision of performing physician who were not informed about the anticoagulation status of AF patients undergoing DCCV. SEC was graded as very mild (minimal echogenicity, only detectable transiently, or increasing gain setting required for the detection), mild (detectable without increasing gain setting), moderate (dense, swirling echogenic material, echogenic signal is dense in LAA compared to LA), or severe (dense, swirling echogenic material, echogenic signal is equivocal in LAA and LA). Dense SEC was defined as a composite of moderate and severe SEC. Representative images of SEC are shown in S1 Fig.

### Statistical analysis

Continuous variables are expressed as means ± standard deviations (SD). Categorical variables are presented as percentile values. Unpaired t-test was used to compare continuous variables. Categorical variables were compared with chi-square test or Fisher’s exact test as appropriate. For multivariate analysis, binary logistic regression analysis was conducted. Univariate analysis was first performed to select appropriate independent variables for inclusion in the multivariate analysis model. Variables with *p* value of less than 0.1 were included. However, individual components of CHA_2_DS_2_-VASc score which are traditional risk factors for ischemic stroke were included in the multivariate model regardless of the results of univariate analysis. Missing data were excluded from each analysis and no imputation was performed. Number of missing data is summarized in S1 Table. All significance tests were two-tailed and *p* values of less than 0.05 were considered statistically significant. All statistical analyses were performed with SPSS version 21.0 (IBM, Armonk, NY, USA).

## Results

### Patient characteristics

**Fig 1:**
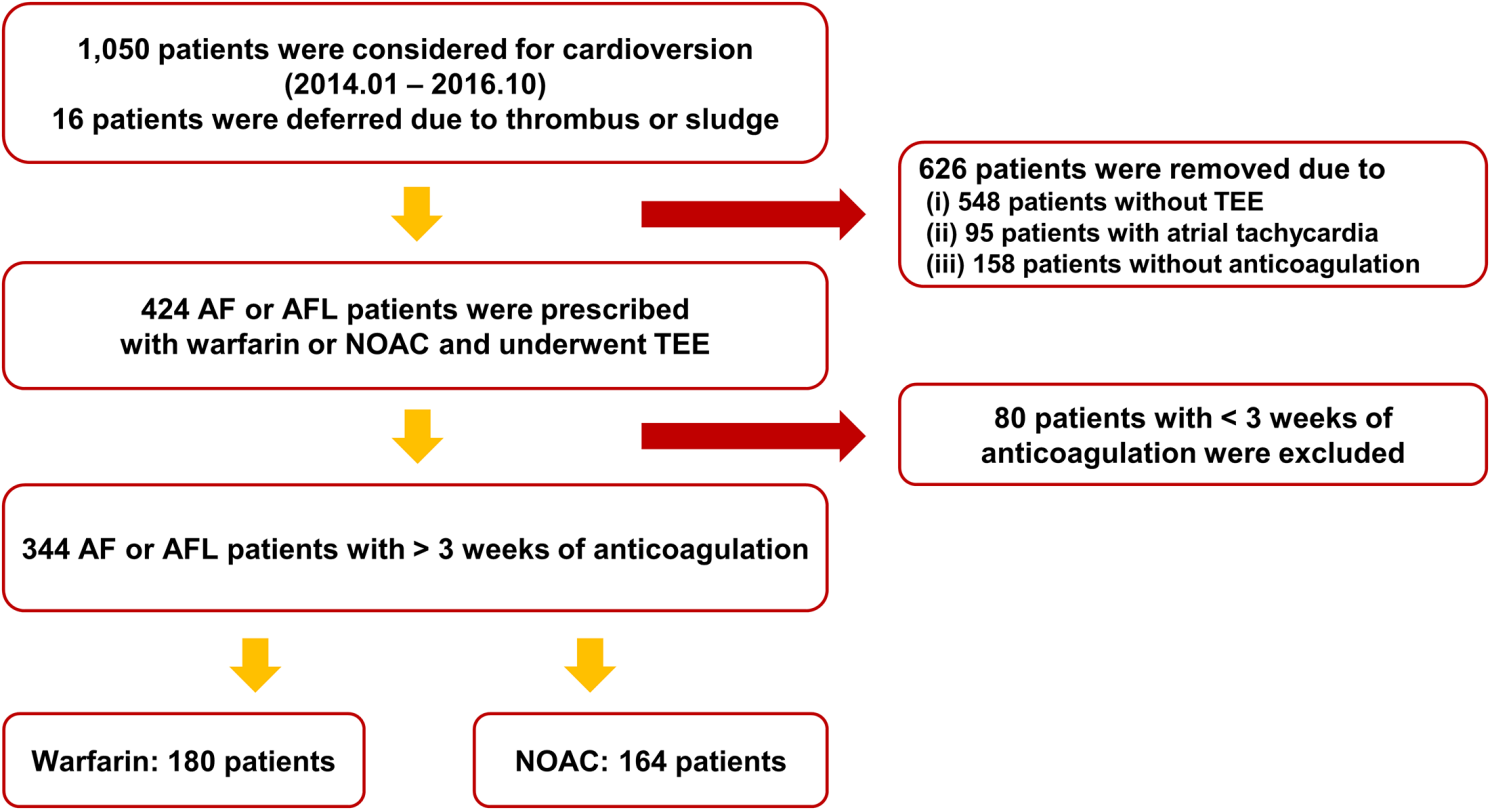
Flow of the study. AF: atrial fibrillation; AFL: atrial flutter; NOAC: non-vitamin K oral anticoagulant; TEE: trans-esophageal echocardiography.

From January 2014 to October 2016, a total of 1,050 patients were considered for electrical DCCV in Korea University Anam Hospital. Among these 1,050 patients, 16 patients did not undergo DCCV due to thrombus observed in LA or LAA in TEE evaluation. However, these 16 patients were included in the analysis if they met the inclusion criteria. Among 1,050 patients, 626 patients were excluded due to lack of TEE evaluation (n = 548), diagnosis other than AF or AFL (n = 95), or absence of anticoagulation therapy (n = 158). Eighty patients were additionally excluded due to inadequate duration (less than 3 weeks) of anticoagulation. Finally, 344 patients (180 patients in the warfarin group and 164 patients in the NOAC group) were included in the analysis (Fig 1). Baseline characteristics of the overall study population, warfarin group, NOAC group, and full dose NOAC group are summarized in Table 1. Mean age of the overall population was 60.0 ± 10.7 years. Of these patients, 77.6% were males. Average CHA_2_DS_2_-VASc score was 1.6 ± 1.3. Baseline characteristics were similar between the warfarin group and the NOAC group. Full dose NOAC group and the warfarin group also had similar baseline characteristics. However, platelet count was significantly higher in the NOAC group compared to that in the warfarin group. The NOAC group also had a statistical tendency for higher CHA_2_DS_2_-VASc score compared to the warfarin group. Full dose NOAC group and warfarin group showed similar CHA_2_DS_2_-VASc scores.

**Table 1:** Baseline characteristics.

	All patients (N = 344)	Warfarin (n = 180)	NOAC (n = 164)	Full dose NOAC (n = 142)	*p* value (Warfarin vs. NOAC)	*p* value (Warfarin vs. Full dose NOAC)
AF	95.1% (327)	93.9% (169)	96.3% (158)	96.5% (137)	0.295	0.288
AFL	4.9% (17)	6.1% (11)	3.7% (6)	3.5 (5)	0.295	0.288
Age	60.0 ± 10.7	60.2 ± 10.0	59.9 ± 11.5	58.3 ± 10.7	0.800	0.110
Male sex	77.6% (267)	80.0% (144)	75.0% (123)	78.9% (112)	0.266	0.804
Body weight (kg)	71.8 ± 12.4	71.8 ± 12.8	71.8 ± 12.1	72.9 ± 11.9	0.989	0.417
Height (cm)	167.5 ± 8.7	167.9 ± 8.5	167.1 ± 8.9	167.9 ± 8.5	0.402	0.994
BMI (kg/m^2^)	25.4 ± 3.1	25.3 ± 3.0	25.6 ± 3.3	25.8 ± 3.3	0.376	0.171
Hypertension	42.7% (147)	38.3% (69)	47.6% (78)	43.0% (61)	0.084	0.401
Diabetes mellitus	11.9% (41)	10.6% (19)	13.4% (22)	12.0% (17)	0.414	0.689
CHF	8.1% (28)	10.0% (18)	6.1% (10)	4.9% (7)	0.186	0.091
Stroke/TIA/SEE	9.9% (34)	8.9% (16)	11.0% (18)	11.3% (16)	0.517	0.479
Vascular disease	2.6% (9)	3.3% (6)	1.8% (3)	1.4% (2)	0.506	0.474
Alcohol	45.2% (154)	47.5% (84)	42.7% (70)	44.4% (63)	0.376	0.582
Smoking	26.8% (91)	24.9% (44)	29.0% (47)	31.4% (44)	0.389	0.195
CHA_2_DS_2_-VASc	1.6 ± 1.3	1.5 ± 1.3	1.7 ± 1.3	1.5 ± 1.2	0.064	0.680
Previous RFCA	16.9% (58)	18.9% (34)	14.6% (24)	14.8% (21)	0.292	0.332
Moderate to severe MR	1.8% (6)	1.7% (3)	1.8% (3)	1.4% (2)	> 0.999	> 0.999
Moderate to severe MS	0.0% (0)	0.0% (0)	0.0% (0)	0.0% (0)		
Moderate to severe AR	0.6% (2)	0.0% (0)	1.2% (2)	0.7% (1)	0.232	0.446
Moderate to severe AS	0.0% (0)	0.0% (0)	0.0% (0)	0.0% (0)		
Mitral valve replacement	0.9% (3)	1.7% (3)	0.0% (0)	0.0% (0)	0.249	0.256
Aortic valve replacement	0.6% (2)	1.1% (2)	0.0% (0)	0.0% (0)	0.499	0.504
Forward LAA flow (cm/sec)	28.9 ± 14.8	28.5 ± 14.9	29.4 ± 14.7	30.4 ± 15.0	0.589	0.270
Backward LAA flow (cm/sec)	27.5 ± 14.2	27.1 ± 14.1	28.0 ± 14.4	28.8 ± 14.6	0.560	0.287
Average LAA flow (cm/sec)	28.2 ± 14.0	27.8 ± 13.8	28.7 ± 14.1	29.6 ± 14.3	0.559	0.258
LA diameter (mm)	45.9 ± 5.8	46.4 ± 6.2	45.4 ± 5.3	45.2 ± 5.2	0.083	0.061
LV EF (%)	50.2 ± 9.1	49.6 ± 9.8	50.8 ± 8.3	50.8 ± 8.4	0.191	0.220
Hemoglobin (g/dL)	14.5 ± 1.6	14.6 ± 1.6	14.4 ± 1.6	14.6 ± 1.5	0.352	0.751
Platelet (10^2^/mm^3^)	204.8 ± 59.6	196.4 ± 64.8	214.5 ± 51.5	212.6 ± 50.0	0.011	0.028
Creatinine (mg/dL)	1.0 ± 0.2	1.0 ± 0.2	1.0 ± 0.2	1.0 ± 0.2	0.141	0.075
INR	2.1 ± 0.8	2.2 ± 0.8	1.4 ± 0.4	1.4 ± 0.4	< 0.001	< 0.001
TTR (%)		48.5 ± 41.5				
Time of INR above 1.8 (%)		59.2 ± 41.8				
Bleeding	0.3% (1)	0.6% (1)	0.0% (0)	0.0% (0)	> 0.999	> 0.999
Type of NOAC						
Rivaroxaban			15.2% (25)	14.8% (21)		
Apixaban			48.8% (80)	51.4% (73)		
Dabigatran			35.4% (58)	33.1% (47)		
Edoxaban			0.6% (1)	0.0% (0)		

AF: atrial fibrillation; AFL: atrial flutter; AR: aortic regurgitation; AS: aortic stenosis; BMI: body mass index; CHF: congestive heart failure; INR: international normalized ratio; MR: mitral regurgitation; MS: mitral stenosis; LA: left atrium; LAA: left atrial appendage; LV EV: left ventricular ejection fraction; NOAC: non-vitamin K antagonist oral anticoagulants; RFCA: radio-frequency catheter ablation; SEE: systemic embolic event; TIA: transient ischemic attack; TTR: time in therapeutic range.

### Warfarin versus NOAC

**Fig 2:**
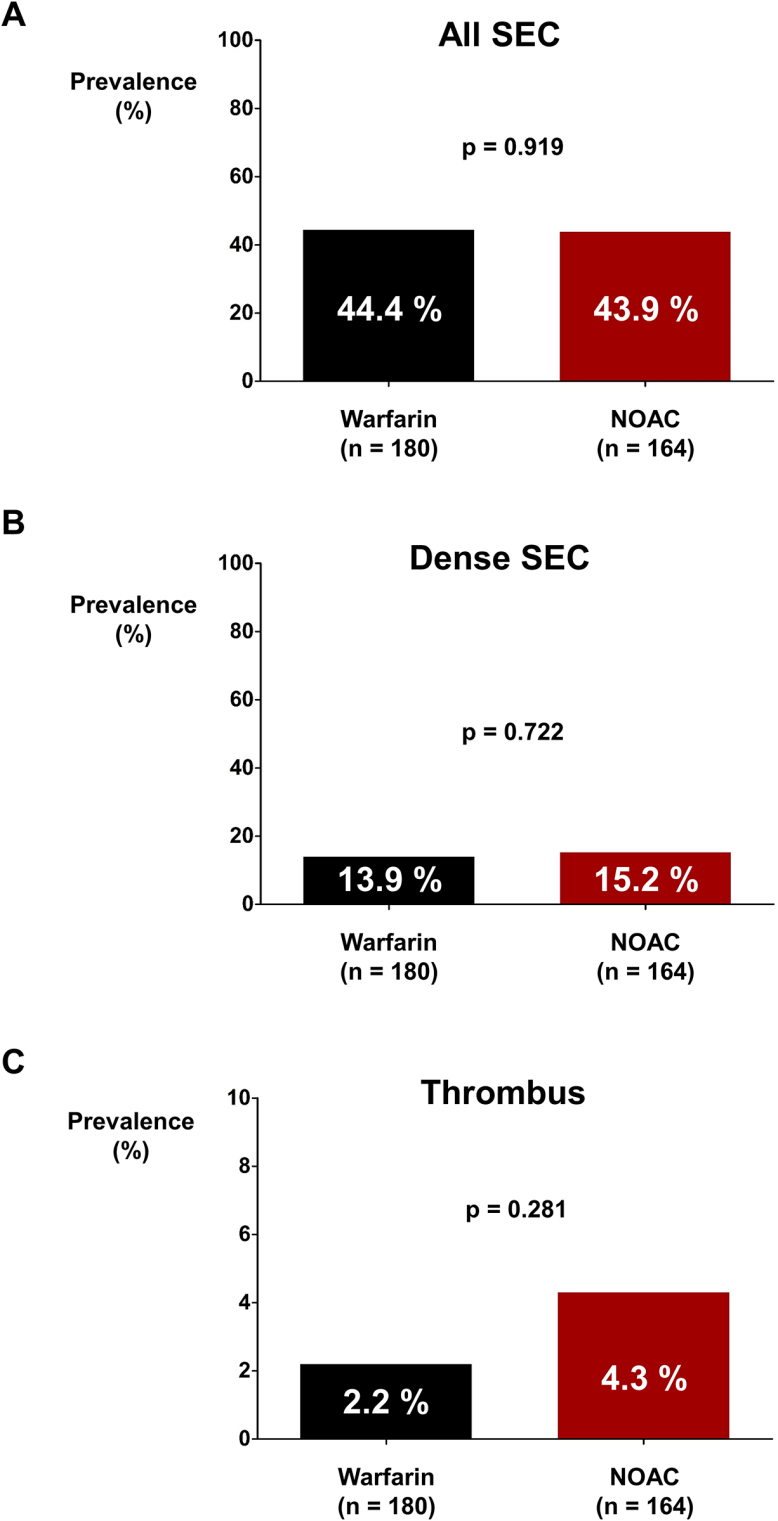
Prevalence rates of SEC and thrombus in warfarin and NOAC groups. Prevalence rates of SEC **(A)**, dense SEC **(B)**, and thrombus **(C)** were not significantly different between warfarin and NOAC groups. NOAC: non-vitamin K oral anticoagulant; SEC: spontaneous echo-contrast.

**Fig 3:**
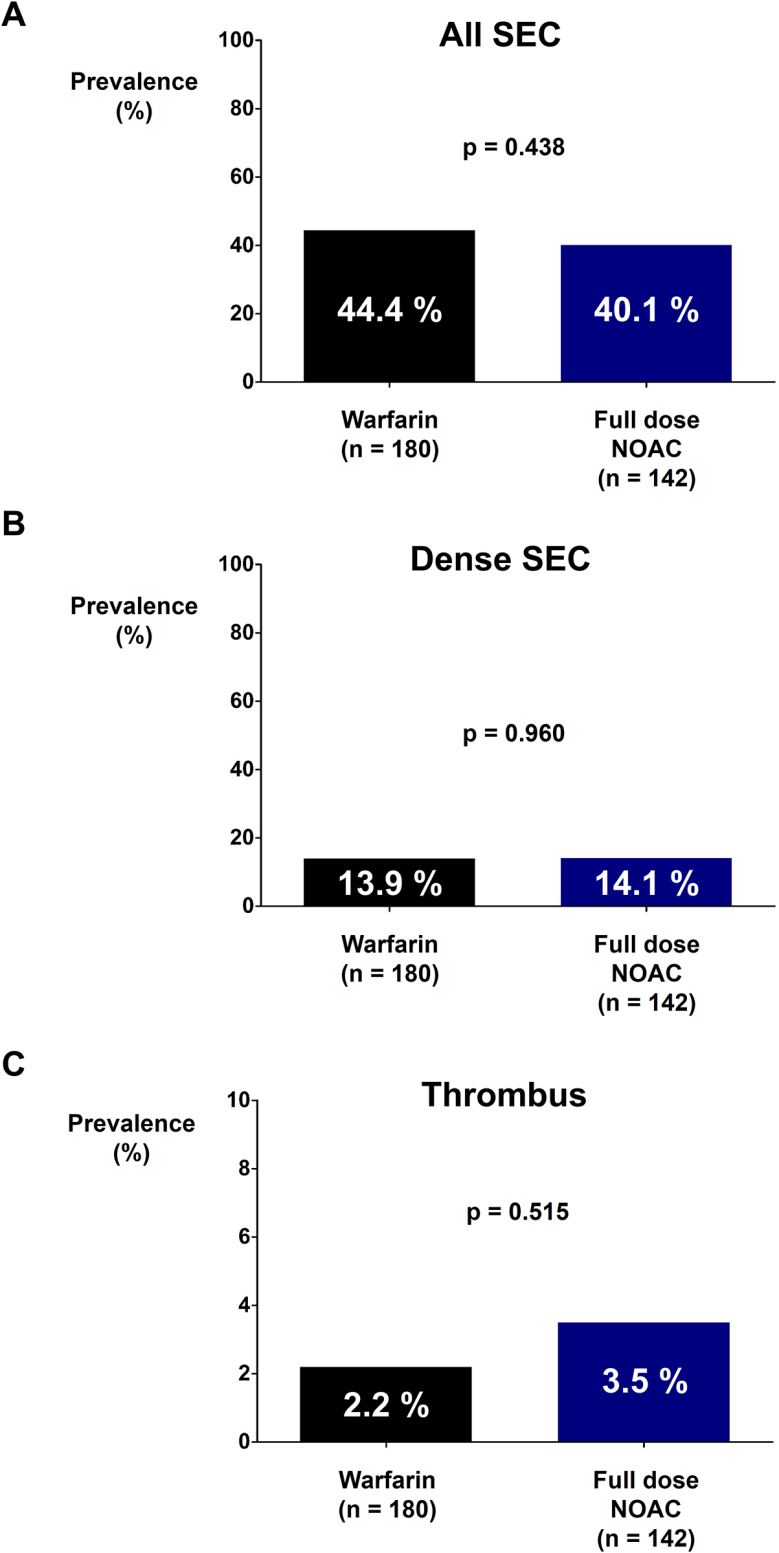
Prevalence rates of SEC and thrombus in warfarin and full dose NOAC groups. Prevalence rates of SEC **(A)**, dense SEC **(B)**, and thrombus **(C)** were not significantly different between warfarin and full dose NOAC groups. NOAC: non-vitamin K oral anticoagulant; SEC: spontaneous echo-contrast.

There were no differences in the prevalence rates of SEC (44.4% vs. 43.9%; *p* = 0.919; Fig 2A) and dense SEC (13.9% vs. 15.2%; *p* = 0.722; Fig 2B) between the warfarin group and the NOAC group. The prevalence rate of thrombus was numerically higher in the NOAC group but without statistical significance (2.2% vs. 4.3%; *p* = 0.281; Fig 2C). The prevalence rates of SEC (44.4% vs. 40.1%; *p* = 0.438; Fig 3A), dense SEC (13.9% vs. 14.1%; *p* = 0.960; Fig 3B), and thrombus (2.2% vs. 3.5%; *p* = 0.515; Fig 3C) were not significantly different between the warfarin group and the full dose NOAC group.

Results of multivariate analysis are shown in Tables 2 and 3. Older Age, increased LA size, decreased LAA flow velocity, and decreased LV ejection fraction were all independently associated with increased prevalence of SEC (Tables 2 and 3). Compared to warfarin, NOAC was not associated with increased or decreased risk of SEC (odds ratio [OR] = 1.352; 95% confidence interval [CI] = 0.796–2.297; *p* = 0.265). The prevalence of thrombus was numerically higher in the NOAC group but it was statistically insignificant (OR = 3.412; 95% CI = 0.726–16.039; *p* = 0.120) (Table 2). Full dose NOAC was also comparable to warfarin regarding the risk of SEC (OR = 1.341; 95% CI = 0.767–2.345; *p* = 0.304) but there was a statistical tendency for increased risk of thrombus formation in LA or LAA (OR = 5.002; 95% CI = 0.801–31.233; *p* = 0.085) (Table 3).

**Table 2:** Multivariate analysis: Warfarin vs. NOAC.

	SEC			Thrombus		
	OR	95% CI	p-value	OR	95% CI	p-value
AF	1.621	0.352–7.468	0.536			
Age	1.043	1.014–1.073	0.003	0.955	0.878–1.040	0.291
Sex	0.821	0.439–1.537	0.538	0.365	0.067–1.976	0.242
CHF	2.374	0.735–7.672	0.149	0.976	0.101–9.474	0.983
Hypertension	1.115	0.642–1.937	0.700	7.301	1.233–43.228	0.028
Diabetes	0.662	0.285–1.538	0.338	2.910	0.590–14.359	0.190
CVA	1.698	0.683–4.218	0.254	7.107	1.180–42.824	0.032
PVD	0.982	0.152–6.359	0.985	3.370	0.186–61.046	0.411
LAA flow velocity	0.920	0.894–0.946	< 0.001	0.881	0.789–0.984	0.025
LA diameter	1.054	1.003–1.107	0.038	1.178	1.019–1.362	0.026
LV EF	0.965	0.936–0.996	0.025	0.945	0.877–1.019	0.144
NOAC	1.352	0.796–2.297	0.265	3.412	0.726–16.039	0.120

**Table 3:** Multivariate analysis: Warfarin vs. full dose NOAC.

	SEC			Thrombus		
	OR	95% CI	p-value	OR	95% CI	p-value
AF	1.774	0.369–8.519	0.474			
Age	1.045	1.014–1.077	0.004	0.963	0.876–1.060	0.444
Sex	0.736	0.376–1.438	0.370	0.380	0.041–3.509	0.393
CHF	2.061	0.604–7.027	0.248	1.310	0.106–16.200	0.833
Hypertension	1.076	0.606–1.910	0.803	10.734	1.237–93.109	0.031
Diabetes	0.544	0.217–1.365	0.195	1.574	0.201–12.334	0.666
CVA	1.657	0.651–4.217	0.289	15.531	1.966–122.675	0.009
PVD	0.876	0.126–6.090	0.893	8.500	0.352–205.180	0.188
LAA flow velocity	0.923	0.897–0.950	0.000	0.881	0.781–0.995	0.042
LA diameter	1.073	1.019–1.130	0.008	1.219	1.025–1.450	0.025
LV EF	0.954	0.923–0.986	0.005	0.910	0.830–0.999	0.047
NOAC	1.341	0.767–2.345	0.304	5.002	0.801–31.233	0.085

AF: atrial fibrillation; CHF: congestive heart failure; CI: confidence interval; CVA: cerebrovascular accident; LA: left atrium; LAA: left atrial appendage; LV EV: left ventricular ejection fraction; NOAC: non-vitamin K antagonist oral anticoagulants; OR: odds ratio; PVD: peripheral vascular disease; SEE: systemic embolic event.

Prevalence rates of SEC, dense SEC, and thrombus between patients with age < 60 and ≥ 60 are presented in S2 Fig. In subgroup analysis, substantial interaction between age and type of anticoagulation (NOAC or warfarin) for having dense SEC was present with patients ≥ 60 years old experiencing higher risk of SEC when treated with NOAC rather than warfarin (p = 0.031; S2 Table). Baseline characteristics of patients with age < 60 and ≥ 60 are presented in S3 Table. Patients with age ≥ 60 had higher CHA_2_DS_2_-VASc score, higher percentage of dose reduction of NOAC, worse TEE findings, and lower body weight. Other subgroup analyses showed no significant interaction between the status of anticoagulation and CHA_2_DS_2_-VASc or LAA flow velocity (S2 Table).

### Impact of dose reduction of NOAC

**Fig 4:**
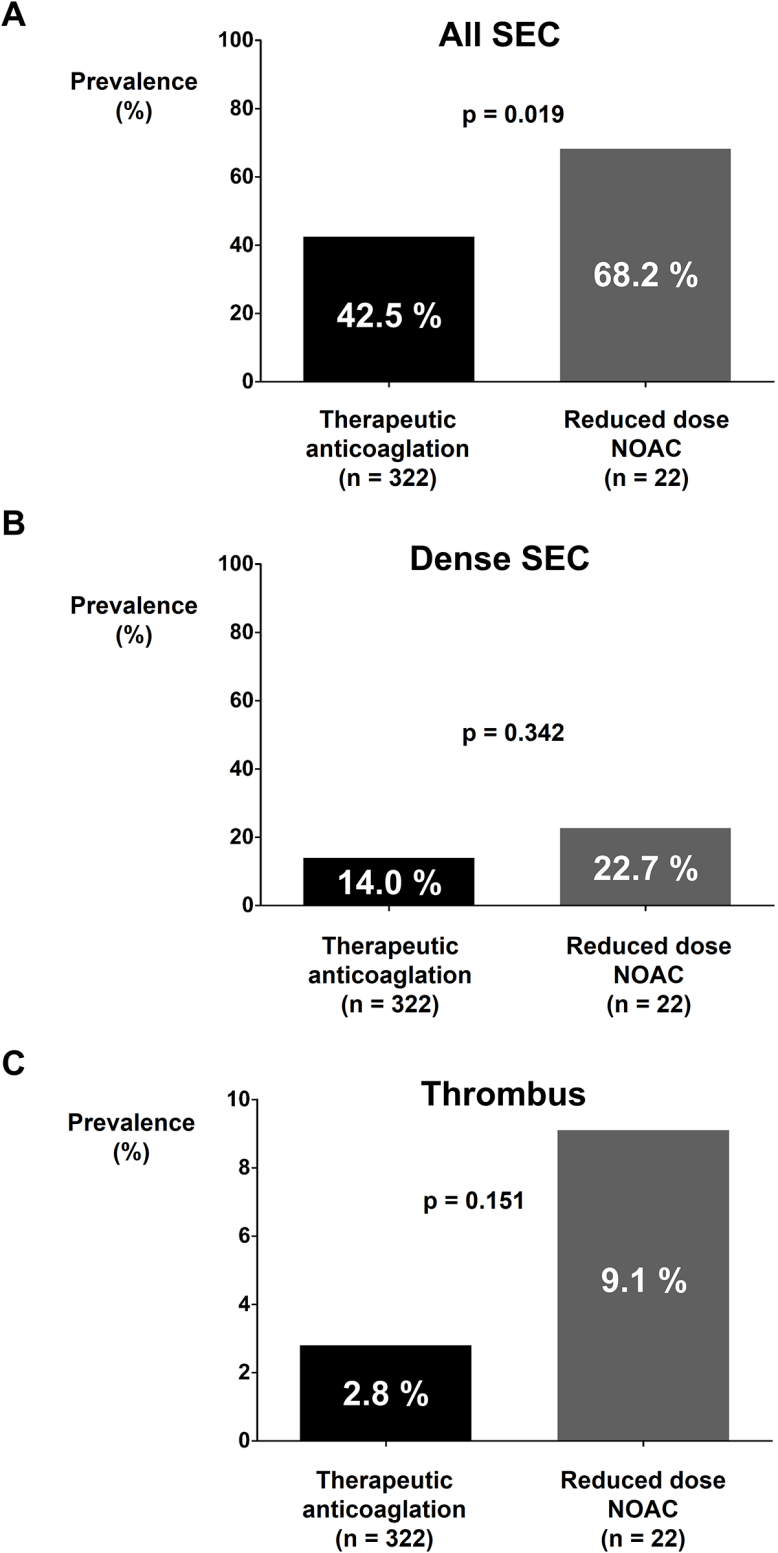
Prevalence rates of SEC and thrombus in therapeutic anticoagulation and reduced dose NOAC groups. The prevalence rate of SEC was significantly higher in the reduced dose NOAC group **(A)**. Prevalence rates of dense SEC **(B)** and thrombus **(C)** were not significantly different between warfarin and reduced dose NOAC groups. NOAC: non-vitamin K oral anticoagulant; SEC: spontaneous echo-contrast.

Among 164 patients treated with NOAC, 22 patients were prescribed with reduced dose of NOAC. The effect of dose reduction of NOAC was evaluated by comparing the prevalence rates of SEC, dense SEC, and thrombus between therapeutic anticoagulation group (warfarin [n = 180] and full dose NOAC [n = 142]; total n = 322) and reduced dose NOAC group (n = 22). Baseline characteristics of patients prescribed with reduced dose of NOAC were significantly different from patients who were prescribed with therapeutic anticoagulation (S4 Table). Patients with reduced dose NOAC had significantly older age, higher rates of female sex and hypertension, lower body weight and height, and higher CHA_2_DS_2_-VASc score compared to patients prescribed with therapeutic anticoagulation. Creatinine level was not significantly different between the two groups. Significant difference in the prevalence rate of SEC was observed between the two groups (42.5% vs. 68.2%; *p* = 0.019; Fig 4A). The prevalence of dense SEC and thrombus was higher in the reduced dose NOAC group compared to that of the therapeutic anticoagulation group. However, the difference was not statistically significant (Fig 4B and 4C).

### Efficacy of each NOAC

**Fig 5:**
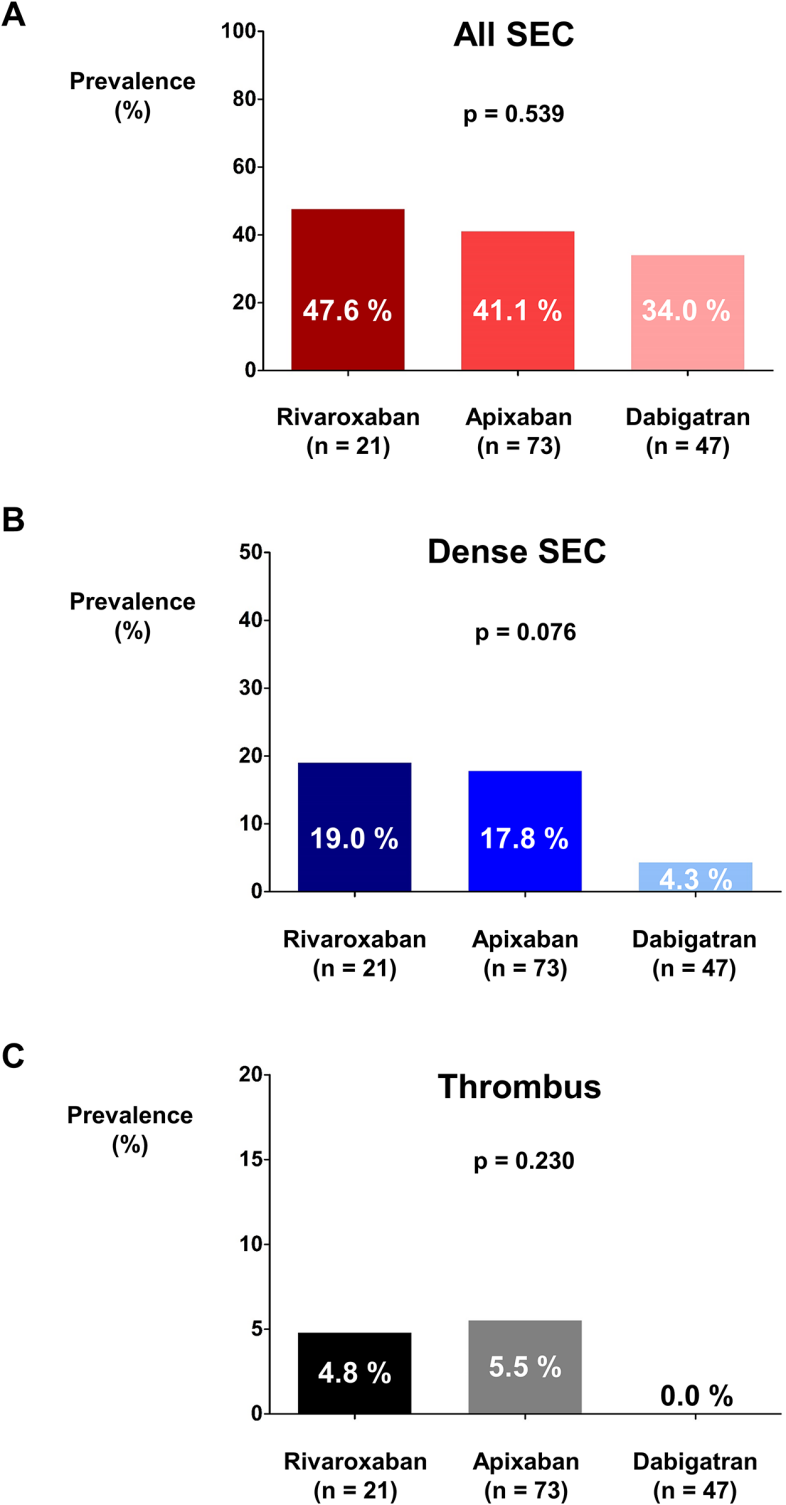
Prevalence rates of SEC, dense SEC, and thrombus among rivaroxaban, apixaban, and dabigatran groups. Prevalence rates of SEC **(A)**, dense SEC **(B)**, and thrombus **(C)** were not significantly different among full dose rivaroxaban, apixaban, and dabigatran groups. NOAC: non-vitamin K oral anticoagulant; SEC: spontaneous echo-contrast.

Among 164 NOAC users, 25, 80, and 58 patients were prescribed with rivaroxaban, apixaban, and dabigatran, respectively. After excluding patients who were prescribed with reduced dose of NOAC, 21, 73, and 47 patients were prescribed with full dose of rivaroxaban, apixaban, and dabigatran, respectively. There were no differences in the prevalence rates of SEC (47.6% vs. 41.1% vs. 34.0%; *p* = 0.539; Fig 5A), dense SEC (19.0% vs. 17.8% vs. 4.3%; *p* = 0.076; Fig 5B), and thrombus (4.8% vs. 5.5% vs. 0.0%; *p* = 0.230; Fig 5C) among NOAC users prescribed with rivaroxaban, apixaban, and dabigatran.

## Discussion

The prescription of NOAC in patients with AF is increasing explosively. NOAC is also gaining its popularity for the prevention of ischemic stroke during peri-DCCV period of AF patients. Therefore, confirming the efficacy of NOAC in preventing thrombotic complication compared to warfarin in AF patients undergoing DCCV is important. Previous studies evaluated the efficacy of NOAC in patients with AF undergoing elective DCCV based on ischemic stroke events which had very low incidence rate to acquire sufficient statistical power to compare the antithrombotic efficacies of warfarin and NOAC. To the best of our knowledge, no study has reported TEE findings of patients with AF undergoing DCCV prescribed with warfarin or NOAC.

The current study only included patients who underwent at least 3 weeks of anticoagulation before undergoing TEE evaluation. Therefore, we were able to compare the steady state effect of warfarin and NOAC. Our results suggest that NOAC and warfarin have similar effects in preventing SEC and dense SEC. Statistically insignificant but numerically higher prevalence rate of thrombus was observed in the NOAC group which is hard to ignore. The reason for this apprehensive finding is not clear. Possible explanations are (i) absence of reliable test to monitor and adjust the dose of NOAC (ii) inadequate dose reduction of NOAC. Dose reduction of NOAC was performed in patients with older age and low body weight as recommended by the current guidelines. However, the prevalence rates of SEC, dense SEC, and thrombus in the reduced dose NOAC group were high and should not be overlooked. Therefore, the efficacy and safety of reducing dose strategy need further validation.

### Fibrin-rich red thrombus: NOAC vs. Warfarin

The underlying mechanism of increased risk of ischemic stroke in AF patients is an area that is being active investigated. One explanation is that blood stasis caused by AF might render LA and LAA as a suitable place for the development of fibrin-rich red thrombus [16, 21, 22]. In contrast to acute coronary syndrome mainly caused by platelet-rich white thrombus, AF related thrombus is known to be fibrin-rich red thrombus [21, 23]. Byproducts of the coagulation cascades such as thrombin/antithrombin III and prothrombin fragment 1+2 are associated with thrombus and SEC observed in LA or LAA [20, 24, 25]. However, the role of platelets in the formation of SEC or thrombus in AF patients is limited [21]. Therefore, the main goal of antithrombotic treatment for patients with AF is to prevent fibrin formation rather than to prevent platelet aggregation [26]. Aspirin and P2Y12 inhibitors widely used to prevent platelet aggregation in patients with coronary artery disease has limited efficacies compared to warfarin for the prevention of ischemic stroke in patients with AF [26, 27] and therapeutic anticoagulation with warfarin has been the standard of care for AF patients to prevent ischemic stroke events. Recently, four major randomized controlled trials [11–14] and its meta-analysis [28] have revealed that dabigatran, rivaroxaban, apixaban, and edoxaban have efficacies equivalent to warfarin in preventing ischemic stroke. Furthermore, NOAC has significant benefit compared to warfarin regarding all-cause death and hemorrhagic stroke [28]. Prospective study (X-RTA) and retrospective registry (CLOT-AF) have demonstrated an acceptable rate of thrombus resolution after treating patients with LA or LAA thrombus with rivaroxaban [29]. However, meta-analysis of reduced dose of NOAC (dabigatran 110 mg twice daily and edoxaban 30 mg once daily) showed increased risk of ischemic stroke and myocardial infarction as compared to warfarin, although it had more profound reduction for the incidence of major bleeding [28]. In general, our data is in accordance with the previous studies. Although limited by a small sample size, our results also suggest that reduced dose NOAC might increase the risk of SEC or thrombus. Our subgroup analysis suggests a potential interaction between age and type of anticoagulation (NOAC or warfarin) regarding the risk of dense SEC with old patients having higher prevalence of dense SEC when prescribed with NOAC. Patients with old age had significantly higher CHA_2_DS_2_-VASc score, worse TEE findings, and higher percentage of dose reduction of NOAC. These unfavorable clinical characteristics of old age patients might have altered the efficacy of NOAC. Therefore, efficacy and safety of NOAC, especially in reduced dose, in specific subgroups such as patients with old age needs further validation in large clinical trials.

The prevalence rates of SEC, dense SEC, and thrombus among each full dose NOAC were not significantly different. However, dabigatran showed numerically lower prevalence rates for SEC, dense SEC, and thrombus compared to rivaroxaban or apixaban. Previous trials indicated that dabigatran might have relatively better efficacy in preventing ischemic stroke and systemic embolism [28]. The relative risk of ischemic stroke or systemic embolism compared to warfarin was 0.66 (95% CI = 0.53–0.82; *p* = 0.0001) for dabigatran [11, 28]. It was 0.88 (95% CI = 0.75–1.03; *p* = 0.12) for rivaroxaban [12, 28] and 0.80 (95% CI = 0.67–0.95; *p* = 0.012) for apixaban [13, 28]. Therefore, our results were generally in accordance with results of previous trials. However, comparison of efficacy of each NOAC needs further evaluation.

In regard of adequacy of warfarin treatment, mean TTR was 48.5% and time of INR above 1.8 was 59.2% as an average in the current study. Our results were in accordance with previous data. In the GARFIELD-AF prospective registry, the mean INR was lower in Asia than in other regions of the world (ORW) (2.0 vs 2.4) [30]. The proportion of time in the therapeutic range, defined as target INR of 2.0–3.0, was substantially lower in Asia (31.1% vs 54.1%) [30]. Considering the narrow therapeutic window of INR, lower TTR and mean INR seems to be appropriate in East Asian patients. Yamashita and his colleagues reported that as compared with Caucasians, effectiveness profile of INR observed in Japanese patients were virtually identical for ischemic stroke and systemic embolism [31]. However, risk profile of INR shifted leftward by approximately 0.5 INR for intracranial hemorrhage which suggests that in East Asian patients [31], target INR of 2.0–3.0 might substantially increase the bleeding risk. Therefore, considering these studies performed in East Asia, the mean TTR and time of INR above 1.8 in the current study are appropriate.

### TEE findings and risk of SEC or thrombus

Currently, TEE is the gold standard for the evaluation of LA or LAA thrombus which is associated with significantly increased risk of future ischemic stroke [32]. Computed tomography (CT) scan is widely used in clinics for AF patients especially when undergoing radiofrequency catheter ablation. However, CT scan have limited role in detecting LA or LAA thrombus. Thrombus in LA or LAA is usually not large enough to be detected by CT scan and especially thrombus located between pectinate muscles can only be found by TEE. TEE have additional advantage over CT scan in detecting SEC and LAA flow velocity which are also known risk factors for ischemic stroke in AF patients [32, 33]. TTE can also help to identify patients at risk of stroke. CHA_2_DS_2_-VASc and CHADS_2_ scores are useful tools for predicting future thromboembolic events [34–36]. However, other TTE parameters such as LA size, LV ejection fraction, and E/E’ are also associated with increased risk of thromboembolism. Kim et al. have reported that adding LA function, LA volume, or LV ejection fraction to CHA_2_DS_2_-VASc or CHADS_2_ scores can improve the predictive value for the presence of thrombus or dense SEC [34]. In our multivariate analysis, LA size, LV ejection fraction, and average LAA flow velocity were independent predictors for the presence of SEC or thrombus. Every 1 cm/sec increase in average LAA flow velocity, 1 mm increase in LA size, and 1% increase in LV ejection fraction were independently associated with 8% decrease, 4.6% increase, and 3.5% decrease in the presence of SEC, respectively. Average LAA flow velocity and LA size were also independent risk factors for the presence of thrombus in LA or LAA.

It is well known that AF begets AF. As AF persists, structural and functional remodeling of LA continues. Subsequently, LA size will continue to enlarge and LAA flow velocity will decrease gradually [34, 37]. LV ejection fraction might also decrease. The shortcoming of warfarin and NOAC is that, despite its ability to prevent ischemic stroke, it cannot prevent LA remodeling. The median follow-up durations of four major trials [11–14] comparing warfarin and NOAC were between 2 and 3 years. This might not be long enough to fully evaluate the long-term antithrombotic effect of these drugs in an environment with ongoing LA and LV remodeling, thus the long-term efficacies of warfarin and NOAC need further validation. Furthermore, previous studies have demonstrated that reverse remodeling of the LA can occur following successful catheter ablation or DCCV of AF [38]. In addition, treatments targeting reverse remodeling may affect thrombogenicity of AF [39]. Therefore, the role of NOAC in patients with successful rhythm control also deserves extensive investigation.

### Limitations

This study has several limitations. First, this was a retrospective analysis and the choice between warfarin and NOAC was based on the discretion of treating physician rather than randomization. Although baseline characteristics were well matched between the two groups, the influence of hidden confounders might still exist. Second, the sample size of the current study might not be large enough to draw a robust conclusion on whether NOAC is associated with increased risk of thrombus formation. Subgroup analyses were also limited due to small sample size. In order to have sufficient statistical power for differentiation of the prevalence of thrombus between two groups, 1,118 and 286 patients for each group were required by assuming between group difference based on Fig 2C and Table 2 respectively. Comparison of ischemic stroke event rate was not possible due to the small sample size. Third, TEE and TTE data before the start of anticoagulation were unavailable.

## Conclusions

This study showed that NOACs were as effective as warfarin in AF or AFL patients undergoing DCCV, which might enable prompt DCCV without time delay until optimization of INR. However, the prevalence rate of thrombus was not low and was numerically higher in the NOAC group, especially with reduced dose, which warrants further validation of NOAC.

## Supporting information

S1 Fig Representative images of SEC. Very mild SEC: minimal echogenicity detected by increasing gain setting;
Mild SEC: minimal echogenicity detected without increasing gain setting;
Moderate SEC: dense, swirling echogenic material which is denser in LAA compared to LA;
Severe SEC: dense, swirling echogenic material with equivocal density in LAA and LA;
Thrombus: definite mass like echogenic material.
All images are obtained from high esophageal two chamber view.
LA: left atrium; LAA: left atrial appendage; SEC: spontaneous echo-contrast.
(TIF)

S2 Fig Efficacy of NOAC compared to warfarin stratified by age. Prevalence rates of SEC, dense SEC, and thrombus for both warfarin and NOAC groups are presented which are stratified by age.
NOAC: non-vitamin K antagonist oral anticoagulants; SEC: spontaneous echo-contrast.
(TIF)

S1 Table Missing data for each variable. (DOCX)

S2 Table Subgroup analysis showing odds ratio of NOAC for having SEC, dense SEC, or thrombus as compared with warfarin. (DOCX)

S3 Table Clinical characteristics: Age < 60 versus age ≥ 60. (DOCX)

S4 Table Baseline characteristics: Therapeutic anticoagulation versus reduced dose NOAC. (DOCX)

## Abbreviations

AF: atrial fibrillation
AFL: atrial flutter
CI: confidence interval
CT: computed tomography
DCCV: direct current cardioversion
INR: international normalized ratio
LA: left atrium
LAA: left atrial appendage
LV: left ventricle
NOAC: non-vitamin K antagonist oral anticoagulants
OR: odds ratio
SD: standard deviation
SEC: spontaneous echo-contrast
TEE: trans-esophageal echocardiography
TTE: trans-thoracic echocardiography
