# Supplementary figures and images for: Non-vitamin K antagonist oral anticoagulants versus warfarin for the prevention of spontaneous echo-contrast and thrombus in patients with atrial fibrillation or flutter undergoing cardioversion: A trans-esophageal echocardiography study
Source: PLoS One. 2018 Jan 23;13(1):e0191648. doi: 10.1371/journal.pone.0191648 (PMC5779688; doi:10.1371/journal.pone.0191648)

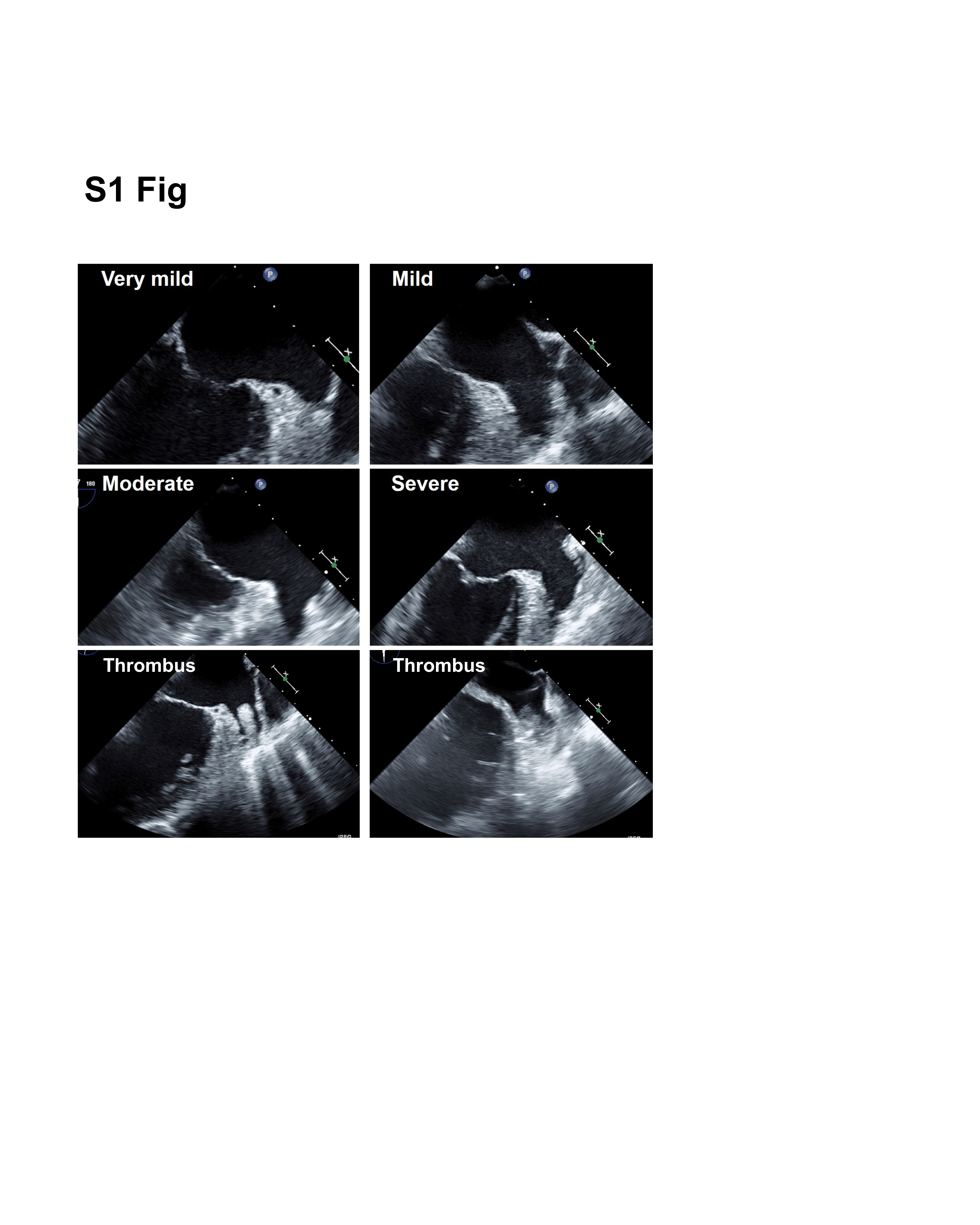

Supplement: S1 Fig — Very mild SEC: minimal echogenicity detected by increasing gain setting; Mild SEC: minimal echogenicity detected without increasing gain setting; Moderate SEC: dense, swirling echogenic material which is denser in LAA compared to LA; Severe SEC: dense, swirling echogenic material with equivocal density in LAA and LA; Thrombus: definite mass like echogenic material. All images are obtained from high esophageal two chamber view. LA: left atrium; LAA: left atrial appendage; SEC: spontaneous echo-contrast. (TIF) [file pone.0191648.s001.tif]

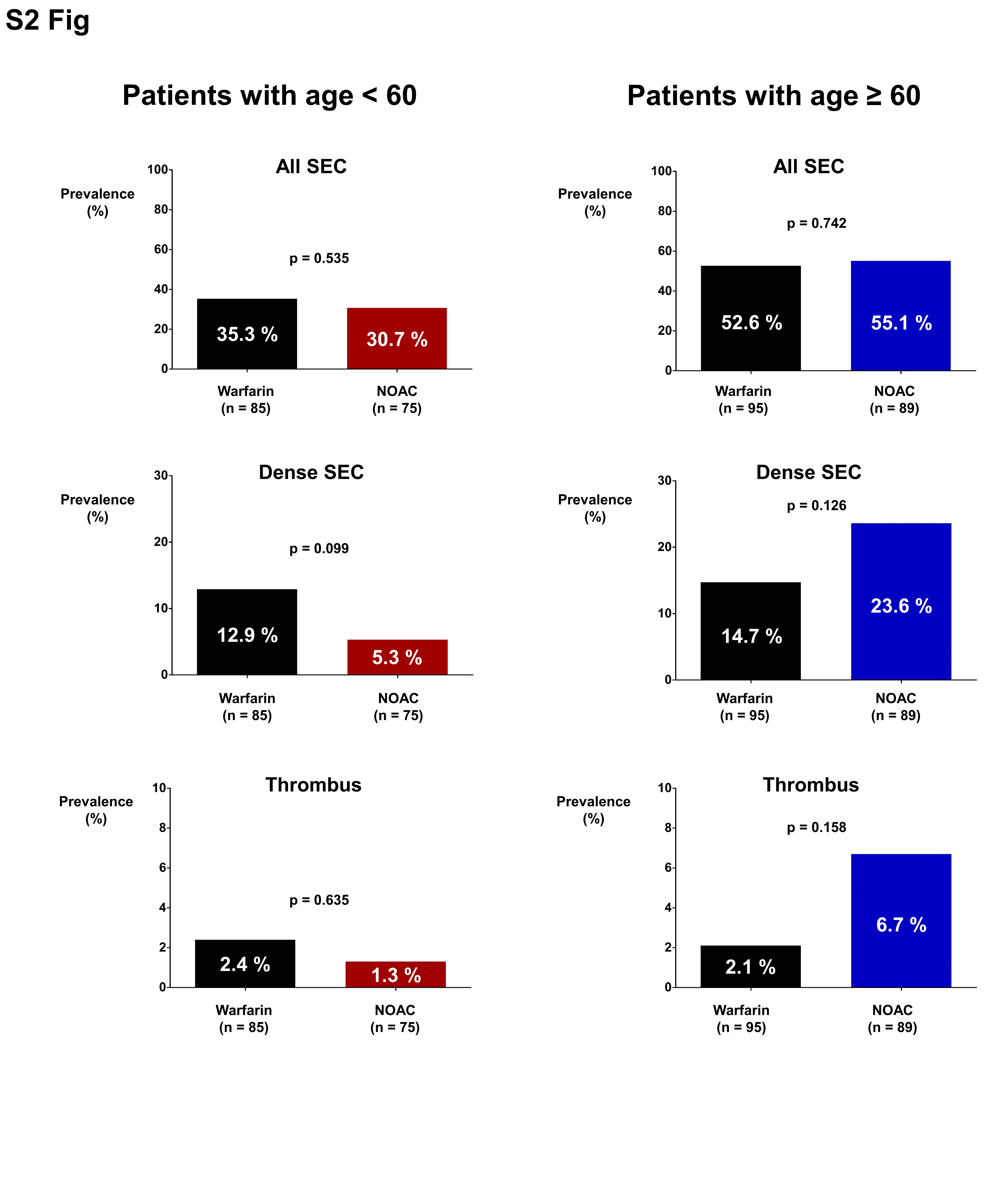

Supplement: S2 Fig — Prevalence rates of SEC, dense SEC, and thrombus for both warfarin and NOAC groups are presented which are stratified by age. NOAC: non-vitamin K antagonist oral anticoagulants; SEC: spontaneous echo-contrast. (TIF) [file pone.0191648.s002.tif]
