# Supplementary material for: Non-vitamin K antagonist oral anticoagulants versus warfarin for the prevention of spontaneous echo-contrast and thrombus in patients with atrial fibrillation or flutter undergoing cardioversion: A trans-esophageal echocardiography study
Source: PLoS One. 2018 Jan 23;13(1):e0191648. doi: 10.1371/journal.pone.0191648 (PMC5779688; doi:10.1371/journal.pone.0191648)
Supplement: S1 Table — (DOCX) [file pone.0191648.s003.docx]

**S1 Table. Missing data for each variable.**

|  | Missing data  (N = 344) | Warfarin  (n = 180) | NOAC  (n = 164) |
| --- | --- | --- | --- |
| AF | None | None | None |
| AFL | None | None | None |
| Age | None | None | None |
| Male sex | None | None | None |
| Body weight (kg) | 6 | 2 | 4 |
| Height (cm) | 6 | 2 | 4 |
| BMI (kg/m^2^) | 6 | 2 | 4 |
| Hypertension | None | None | None |
| Diabetes mellitus | None | None | None |
| CHF | None | None | None |
| Stroke/TIA/SEE | None | None | None |
| Vascular disease | None | None | None |
| Alcohol | 3 | 3 | None |
| Smoking | 5 | 3 | 2 |
| CHA_2_DS_2_-VASc | None | None | None |
| Previous RFCA | None | None | None |
| Moderate to severe MR | None | None | None |
| Moderate to severe MS | None | None | None |
| Moderate to severe AR | None | None | None |
| Moderate to severe AS | None | None | None |
| Mitral valve replacement | None | None | None |
| Aortic valve replacement | None | None | None |
| Forward LAA flow (cm/sec) | 1 | 1 | 0 |
| Backward LAA flow (cm/sec) | 1 | 1 | 0 |
| Average LAA flow (cm/sec) | 1 | 1 | 0 |
| LA diameter (mm) | 7 | 6 | 1 |
| LV EF (%) | 7 | 6 | 1 |
| Hemoglobin (g/dL) | 63 | 30 | 33 |
| Platelet (10^2^/mm^3^) | 64 | 30 | 34 |
| Creatinine (mg/dL) | 64 | 30 | 34 |
| INR | 197 | 1 | 146 |
| Bleeding | None | None | None |
| Type of NOAC |  |  |  |
| Rivaroxaban | None | None | None |
| Apixaban | None | None | None |
| Dabigatran | None | None | None |
| Edoxaban | None | None | None |

AF: atrial fibrillation; AFL: atrial flutter; AR: aortic regurgitation; AS: aortic stenosis; BMI: body mass index; CHF: congestive heart failure; INR: international normalized ratio; MR: mitral regurgitation; MS: mitral stenosis; LA: left atrium; LAA: left atrial appendage; LV EV: left ventricular ejection fraction; NOAC: non-vitamin K antagonist oral anticoagulants; RFCA: radio-frequency catheter ablation; SEE: systemic embolic event; TIA: transient ischemic attack.
