# Supplementary material for: Non-vitamin K antagonist oral anticoagulants versus warfarin for the prevention of spontaneous echo-contrast and thrombus in patients with atrial fibrillation or flutter undergoing cardioversion: A trans-esophageal echocardiography study
Source: PLoS One. 2018 Jan 23;13(1):e0191648. doi: 10.1371/journal.pone.0191648 (PMC5779688; doi:10.1371/journal.pone.0191648)
Supplement: S2 Table — (DOCX) [file pone.0191648.s004.docx]

**S2 Table. Subgroup analysis showing odds ratio of NOAC for having SEC, dense SEC, or thrombus as compared with warfarin.**

**(A)** High CHA_2_DS_2_-VASc vs. low CHA_2_DS_2_-VASc

|  | **Subgroups** | **OR of NOAC for having SEC, dense SEC, or thrombus** | | | **p value** | **p for interaction** |
| --- | --- | --- | --- | --- | --- | --- |
| **SEC** | CHA_2_DS_2_-VASc ≥ 2 | 0.972 (0.527 – 1.792) | | | 0.928 | 0.833 |
|  | CHA_2_DS_2_-VASc 0 – 1 | 0.886 (0.481 – 1.632) | | | 0.697 |  |
|  |  |  |  |  |  |  |
| **Dense SEC** | CHA_2_DS_2_-VASc ≥ 2 | 1.011 (0.475 – 2.152) | | | 0.978 | 0.973 |
|  | CHA_2_DS_2_-VASc 0 – 1 | 1.033 (0.367 – 2.910) | | | 0.951 |  |
|  |  |  |  |  |  |  |
| **Thrombus** | CHA_2_DS_2_-VASc ≥ 2 | 1.852 (0.447 – 7.669) | | | 0.395 | 0.835 |
|  | CHA_2_DS_2_-VASc 0 – 1 | 1.329 (0.082 – 21.588) | | | 0.842 |  |
|  |  |  |  |  |  |  |

**(B)** Age ≥ 60 vs. age < 60

|  | **Subgroups** | **OR of NOAC for having SEC, dense SEC, or thrombus** | | | **p value** | **p for interaction** |
| --- | --- | --- | --- | --- | --- | --- |
| **SEC** | Age ≥ 60 | 1.102 (0.617 – 1.969) | | | 0.742 | 0.494 |
|  | Age < 60 | 0.811 (0.418 – 1.573) | | | 0.535 |  |
|  |  |  |  |  |  |  |
| **Dense SEC** | Age ≥ 60 | 1.787 (0.845 – 3.779) | | | 0.129 | 0.031 |
|  | Age < 60 | 0.379 (0.115 – 1.246) | | | 0.110 |  |
|  |  |  |  |  |  |  |
| **Thrombus** | Age ≥ 60 | 3.361 (0.660 – 17.113) | | | 0.144 | 0.229 |
|  | Age < 60 | 0.561 (0.050 – 6.312) | | | 0.640 |  |
|  |  |  |  |  |  |  |

**(C)** Average LAA flow velocity ≤ 25 cm/sec vs. average LAA flow velocity > 25 cm/sec

|  | **Subgroups** | **OR of NOAC for having SEC, dense SEC, or thrombus** | | | **p value** | **p for interaction** |
| --- | --- | --- | --- | --- | --- | --- |
| **SEC** | LAA flow velocity ≤ 25 | 1.682 (0.647 – 4.369) | | | 0.286 | 0.357 |
|  | LAA flow velocity > 25 | 1.000 (0.573 – 1.747) | | | > 0.999 |  |
|  |  |  |  |  |  |  |
| **Dense SEC** | LAA flow velocity ≤ 25 | 1.310 (0.582 – 2.950) | | | 0.514 | 0.959 |
|  | LAA flow velocity > 25 | 1.357 (0.456 – 4.039) | | | 0.583 |  |
|  |  |  |  |  |  |  |
| **Thrombus** | LAA flow velocity ≤ 25 | 2.375 (0.537 – 10.510) | | | 0.254 | 0.910 |
|  | LAA flow velocity > 25 | 2.017 (0.180 – 22.549) | | | 0.569 |  |
|  |  |  |  |  |  |  |

LAA: left atrial appendage; NOAC: non-vitamin K antagonist oral anticoagulants; OR: odds ratio; SEC: spontaneous echo-contrast.
