# Supplementary material for: Non-vitamin K antagonist oral anticoagulants versus warfarin for the prevention of spontaneous echo-contrast and thrombus in patients with atrial fibrillation or flutter undergoing cardioversion: A trans-esophageal echocardiography study
Source: PLoS One. 2018 Jan 23;13(1):e0191648. doi: 10.1371/journal.pone.0191648 (PMC5779688; doi:10.1371/journal.pone.0191648)
Supplement: S3 Table — (DOCX) [file pone.0191648.s005.docx]

**S3 Table. Clinical characteristics: age < 60 versus age ≥ 60.**

|  | Age < 60  (n = 160) | Age ≥ 60  (n = 184) | p-value |
| --- | --- | --- | --- |
| AF | 155 (96.9%) | 172 (93.5%) | 0.147 |
| AFL | 5 (3.1%) | 12 (6.5%) | 0.147 |
| Age | 51.0 ± 6.9 | 68.0 ± 6.2 | < 0.001 |
| Male sex | 142 (88.8%) | 125 (67.9%) | < 0.001 |
| Body weight (kg) | 76.3 ± 12.6 | 67.8 ± 10.8 | < 0.001 |
| Height (cm) | 170.6 ± 7.9 | 164.9 ± 8.5 | < 0.001 |
| BMI (kg/m^2^) | 26.1 ± 3.3 | 24.8 ± 2.8 | < 0.001 |
| HTN | 45 (28.1%) | 102 (55.4%) | < 0.001 |
| DM | 20 (12.5%) | 21 (11.4%) | 0.756 |
| CHF | 15 (9.4%) | 13 (7.1%) | 0.435 |
| Stroke/TIA/SEE | 16 (10.0%) | 18 (9.8%) | 0.946 |
| Vascular disease | 4 (2.5%) | 5 (2.7%) | 0.900 |
| Alcohol | 95 (59.4%) | 59 (32.6%) | < 0.001 |
| Smoking | 60 (37.7%) | 31 (17.2%) | < 0.001 |
| CHA_2_DS_2_-VASc | 0.9 ± 1.0 | 2.2 ± 1.3 | < 0.001 |
| Previous RFCA | 27 (16.9%) | 31 (16.8%) | 0.995 |
| Forward LAA flow (cm/sec) | 31.4 ± 15.4 | 26.9 ± 13.9 | 0.005 |
| Backward LAA flow (cm/sec) | 29.9 ± 13.7 | 25.4 ± 14.4 | 0.003 |
| Average LAA flow (cm/sec) | 30.6 ± 13.9 | 26.1 ± 13.7 | 0.003 |
| LA diameter (mm) | 45.6 ± 5.9 | 46.2 ± 5.7 | 0.295 |
| LV EF (%) | 49.4 ± 9.1 | 50.9 ± 9.1 | 0.144 |
| PAP (mmHg) | 30.4 ± 6.0 | 32.8 ± 7.2 | 0.002 |
| Hemoglobin (g/dL) | 15.0 ± 1.4 | 14.1 ± 1.7 | < 0.001 |
| Platelet (10^2^/mm^3^) | 206.2 ± 50.8 | 203.5 ± 66.7 | 0.708 |
| Creatinine (mg/dL) | 1.0 ± 0.2 | 1.0 ± 0.2 | 0.396 |
| Dose reduction of NOAC | 4 (5.3%) | 18 (20.2%) | 0.005 |

AF: atrial fibrillation; AFL: atrial flutter; AR: aortic regurgitation; AS: aortic stenosis; BMI: body mass index; CHF: congestive heart failure; INR: international normalized ratio; MR: mitral regurgitation; MS: mitral stenosis; LA: left atrium; LAA: left atrial appendage; LV EV: left ventricular ejection fraction; NOAC: non-vitamin K antagonist oral anticoagulants; PAP: pulmonary artery pressure; RFCA: radio-frequency catheter ablation; SEE: systemic embolic event; TIA: transient ischemic attack.
