# Supplementary material for: Non-vitamin K antagonist oral anticoagulants versus warfarin for the prevention of spontaneous echo-contrast and thrombus in patients with atrial fibrillation or flutter undergoing cardioversion: A trans-esophageal echocardiography study
Source: PLoS One. 2018 Jan 23;13(1):e0191648. doi: 10.1371/journal.pone.0191648 (PMC5779688; doi:10.1371/journal.pone.0191648)
Supplement: S4 Table — (DOCX) [file pone.0191648.s006.docx]

**S4 Table. Baseline characteristics: therapeutic anticoagulation versus reduced dose NOAC.**

|  | All patients  (N = 344) | Therapeutic anticoagulation  (n = 322) | Reduced dose NOAC  (n = 22) | p-value |
| --- | --- | --- | --- | --- |
| AF | 95.1% (327) | 95.0% (306) | 95.5% (21) | > 0.999 |
| AFL | 4.9% (17) | 5.0% (16) | 4.5% (1) | > 0.999 |
| Age | 60.0 ± 10.7 | 59.4 ± 10.3 | 70.0 ± 11.4 | < 0.001 |
| Male sex | 77.6% (267) | 79.5% (256) | 50.0% (11) | 0.003 |
| Body weight (kg) | 71.8 ± 12.4 | 72.3 ± 12.4 | 64.1 ± 11.1 | 0.003 |
| Height (cm) | 167.5 ± 8.7 | 167.9 ± 8.5 | 161.8 ± 9.9 | 0.002 |
| BMI (kg/m^2^) | 25.4 ± 3.1 | 25.5 ± 3.1 | 24.4 ± 3.0 | 0.111 |
| HTN | 42.7% (147) | 40.4% (130) | 77.3% (17) | 0.001 |
| DM | 11.9% (41) | 11.2% (36) | 22.7% (5) | 0.162 |
| CHF | 8.1% (28) | 7.8% (25) | 13.6% (3) | 0.407 |
| Stroke/TIA/SEE | 9.9% (34) | 9.9% (32) | 9.1% (2) | > 0.999 |
| Vascular disease | 2.6% (9) | 2.5% (8) | 4.5% (1) | 0.452 |
| Alcohol | 45.2% (154) | 46.1% (147) | 31.8% (7) | 0.194 |
| Smoking | 26.8% (91) | 27.8% (88) | 13.6% (3) | 0.148 |
| CHA_2_DS_2_-VASc | 1.6 ± 1.3 | 1.5 ± 1.3 | 3.1 ± 1.3 | < 0.001 |
| Previous RFCA | 16.9% (58) | 17.1% (55) | 13.6% (3) | > 0.999 |
| Moderate to severe MR | 1.8% (6) | 1.6% (5) | 4.5% (1) | 0.334 |
| Moderate to severe MS | 0.0% (0) | 0.0% (0) | 0.0% (0) |  |
| Moderate to severe AR | 0.6% (2) | 0.3% (1) | 4.5% (1) | 0.126 |
| Moderate to severe AS | 0.0% (0) | 0.0% (0) | 0.0% (0) |  |
| Mitral valve replacement | 0.9% (3) | 0.9% (3) | 0.0% (0) | > 0.999 |
| Aortic valve replacement | 0.6% (2) | 0.6% (2) | 0.0% (0) | > 0.999 |
| Forward LAA flow (cm/sec) | 28.9 ± 14.8 | 29.4 ± 14.9 | 23.0 ± 11.1 | 0.051 |
| Backward LAA flow (cm/sec) | 27.5 ± 14.2 | 27.8 ± 14.3 | 22.7 ± 12.6 | 0.100 |
| Average LAA flow (cm/sec) | 28.2 ± 14.0 | 28.6 ± 14.0 | 22.8 ± 11.4 | 0.061 |
| LA diameter (mm) | 45.9 ± 5.8 | 45.9 ± 5.8 | 46.1 ± 5.7 | 0.889 |
| LV EF (%) | 50.2 ± 9.1 | 50.1 ± 9.2 | 50.9 ± 8.0 | 0.690 |
| PAP (mmHg) | 31.7 ± 6.8 | 31.5 ± 6.4 | 34.4 ± 10.8 | 0.064 |
| Hemoglobin (g/dL) | 14.5 ± 1.6 | 14.6 ± 1.5 | 12.8 ± 1.8 | < 0.001 |
| Platelet (10^2^/mm^3^) | 204.8 ± 59.6 | 203.3 ± 59.4 | 227.2 ± 60.4 | 0.110 |
| Creatinine (mg/dL) | 1.0 ± 0.2 | 1.0 ± 0.2 | 1.0 ± 0.3 | 0.614 |
| Bleeding | 0.3% (1) | 0.3% (1) | 0.0% (0) | > 0.999 |

AF: atrial fibrillation; AFL: atrial flutter; AR: aortic regurgitation; AS: aortic stenosis; BMI: body mass index; CHF: congestive heart failure; INR: international normalized ratio; MR: mitral regurgitation; MS: mitral stenosis; LA: left atrium; LAA: left atrial appendage; LV EV: left ventricular ejection fraction; NOAC: non-vitamin K antagonist oral anticoagulants; PAP: pulmonary artery pressure; RFCA: radio-frequency catheter ablation; SEE: systemic embolic event; TIA: transient ischemic attack.
